# When responsive and reactive meet organic? Treatment implications of language use in the era of #BanBPSD

**DOI:** 10.1002/gps.5545

**Published:** 2021-04-05

**Authors:** Stephen Macfarlane, Mustafa Atee, Thomas Morris, Colm Cunningham

**Affiliations:** ^1^ The Dementia Centre HammondCare St Leonards New South Wales Australia; ^2^ Faculty of Medicine, Nursing & Health Sciences Monash University Clayton Victoria Australia; ^3^ The Dementia Centre HammondCare Wembley Western Australia Australia; ^4^ Curtin Medical School Faculty of Health Sciences Curtin University Bentley Western Australia Australia; ^5^ School of Public Health & Community Medicine University of New South Wales Sydney New South Wales Australia

**Keywords:** antidepressants, antipsychotics, benefits, BPSD, dementia, (de‐)prescribing, organic aetiology, psychotropics, risks

## Abstract

- The aetiopathogenesis of behaviours and psychological symptoms of dementia (BPSD) is often subjective, complex and multifaceted, produced by an array of contributing factors, including biomedical, psychological, environmental and/or social factors
- Alongside other contributing factors, organic aetiology of BPSD should be considered when devising therapeutic management plans
- Although considered last resort, time‐limited antipsychotic treatment (≤3 months) may have a vital adjunct role in managing intractable, refractory, distressing and/or life‐threatening BPSD, such as delusions and hallucinations; but only after person‐centred psychosocial interventions are exhausted and fail to deliver any therapeutic response
- If prescribed, careful monitoring of therapeutic responses and adverse effects of antipsychotics with de‐prescribing plans should be a top priority, as these agents have limited efficacies and serious adverse outcomes (e.g., mortality)

## 1 BPSD—UNMET, REACTIVE AND/OR ORGANIC?

Debate rages about the extent to which behavioural changes in the context of dementia represent either the consequences of brain changes as the disease progresses, versus a reasonable response to unmet needs. Are these behaviours responsive, reactive or organic?

The #BanBPSD movement appears to emphasise the former, in the main it would seem, as a laudable attempt to reduce the inappropriate use of psychotropic medications for the treatment of behaviours and psychological symptoms of dementia (BPSD).

It is undisputed that person‐centred psychosocial interventions remain the gold standard in supporting BPSD,^1^ but there remains a vast over‐reliance on psychotropic medications for its management. This is despite the fact that these agents have limited efficacy and serious adverse effects. We do, however, contend that psychotropics, including antipsychotics, have a vital role in the management of certain symptoms that would clearly, in our view, seem less interactive with the environment than others, and are better explained as arising from extensive organic changes that accompany a neurodegenerative process.

The contention that BPSD represent a reaction to either unmet needs (e.g., pain),^2^ or change in the environment (e.g., over‐ or under‐stimulation),^3^ is relatively easy to support in most cases.^1^ We accept that stress may arise when the environment places demands upon an individual that require an adaptive response. If an individual's capacity to engage an adaptive response is affected by cognitive impairment, behaviours and psychological manifestations of stress may result. Given the impaired capacity of the individual to change their own response, the alternative is that the ‘environment’ (defined loosely to include not only the physical environment, but also the approach of carers) must adapt to meet the needs of the person(s) living with dementia (PLWD).

To view all behaviours through this lens, however, is to deny the important reality that dementia, in all its forms, is associated with neurodegeneration and altered brain function. As such, it is impossible to deny that disordered brain and neurotransmitter function must underlie a proportion of altered behaviours in this context.

Herein, we present a case for the organicity of certain behaviours, by no means as a defence against the overuse of antipsychotics and other psychotropic drugs (e.g., benzodiazepines), but as a means of highlighting the importance of considering time‐limited psychotropic medications as an adjunct (but last resort) therapy to a holistic model of care for PLWD. ‘Holistic’, as the term is commonly understood, refers to the biopsychosocial model of care. We simply contend, therefore, that biopsychosocial cannot disregard the ‘bio’.

## 2 BPSD: contributing factors and treatment considerations

The aetiopathogenesis of BPSD is often subjective, multifaceted and complex, produced by an array of contributing factors, including biomedical (e.g., brain changes, disease burden, polypharmacy), psychological (e.g., life history, personality traits), environmental (e.g., under‐ or over‐stimulation) and/or social (e.g., family or caregiver support, accommodation and needs) factors.^4^, ^5^, ^6^

Although it is well recognised that environmental factors are important triggers of BPSD,^5^, ^6^, ^7^ the extent that these factors influence the presence and persistence of BPSD are unknown. A review by Van der Linde et al. 2016 investigated the longitudinal effect of various factors (e.g., medication use) on the presence and persistence of BPSD.^8^ However, the review did not study the longitudinal impact of environment on the presence and persistence of BPSD because these factors are difficult to measure.^8^ Studying the longitudinal impact of these covariates could improve our understanding of the potential mechanisms involved in the presence and persistence of BPSD.

Due to the complexity of BPSD, the best therapeutic approach should involve a comprehensive aetiopathogenetic assessment followed initially by multimodal person‐centred psychosocial/non‐pharmacological interventions—as these are deemed the current gold standard. A recent study by Macfarlane et al. (2021) demonstrated that there were significant improvements in neuropsychiatric outcomes (measured by the Neuropsychiatric Inventory [NPI]) associated with the provision of psychosocial person‐centred care interventions delivered by national multidisciplinary dementia‐specific behaviour support programs.^9^ In fact, total NPI scores were reduced by 61.4% and 74.3%, whilst NPI distress scores were reduced by 66.5% and 69.1% for the Dementia Behaviour Management Advisory Service (DBMAS) and the Severe Behaviour Response Team (SBRT) programs, respectively.^9^

Psychotropic medications should only be trialed in PLWD if multimodal non‐pharmacological interventions fail to deliver the desired therapeutic outcome and only when these intervention options are exhausted. That is, psychotropic medications should be prescribed exclusively in cases where it is inevitable otherwise to use such agents.^4^ Due to the severity of adverse effects of antipsychotics, it is always prudent to weigh up the benefits versus potential risks before considering the prescribing of these agents. In all instances, antipsychotics should be reserved for refractory and intractable cases, where these agents can confer the individual a window of opportunity to live life again with some human decency, while non‐pharmacological options are completely exhausted. In such instances, the most effective agent that targets the serious symptoms with the least side effect profile for the shortest period possible should be carefully selected. These considerations need to be discussed with caregivers and/or family members by the treating physician prior to making the decision to initiate antipsychotic treatment. Consent for treating the patient should also be sought from a legal guardian or next of kin.

Although, there is a lack of evidence regarding prolonged use, antipsychotic treatment initiated for BPSD is often inappropriately continued beyond the maximum recommended duration (i.e., 3 months) for managing these symptoms.^10^ In some cases, sadly, prescribing these agents could even become a permanent rather than a rescue solution, causing serious harm to the individual. Because BPSD fluctuate or dissipate over time as the disease progresses,^11^ it is crucial to re‐evaluate the continued need for such treatment and cease treatment as soon as practical—whether the desired therapeutic outcomes (e.g., behaviours stabilised) are achieved or not; or if signs of serious adverse effects (e.g., delirium) become apparent (whichever happens sooner).^12^, ^13^ It is important to note that de‐prescribing plans (e.g., scheduled dose reduction or discontinuation) should be in place when embarking on prescribing any psychotropic medication for PLWD.^13^

## 3 ORGANICITY AND TREATMENT OF PSYCHOTIC SYMPTOMS

The occurrence of distressing psychotic symptoms is perhaps the most obvious area where antipsychotics have an important role in improving quality of life for PLWD. Psychiatrists understand the term psychosis to cover three broad domains; delusions, hallucinations and ‘formal thought disorder’ (literally, a disorder in the ‘form’ of thought that manifests as disturbed thinking and disordered speech). We should exclude formal thought disorder in the context of dementia, as disturbed thinking and disordered speech are invariable as the condition progresses and are clearly not psychotically‐mediated in the presence of neurodegenerative disease and significantly impaired cognition.

Delusions can be defined as fixed, false beliefs that are not amenable to modification by the presentation of evidence to the contrary. We find this definition problematic in the context of dementia as many ‘delusional’ beliefs that are commonly expressed by PLWD (e.g., ‘delusions of theft’) are better explained as arising from artefacts of memory impairment.

The use of the term delusion in this context also raises the expectation that the symptom will respond to an antipsychotic. It is reasonable to suspect that much inappropriate prescribing of antipsychotics occurs in the vain hope that such beliefs will be antipsychotic‐responsive. This is not to say that bizarre psychotic delusions do not occur in the setting of dementia. They clearly do, and when these are causing serious distress or life is threatened it is quite reasonable to use an antipsychotic for their treatment.

**Case example:** Joan is an 83‐year old living at home with early Alzheimer's dementia. Despite having no real insight into her memory impairments, she remains very functional within the home, and is independent apart from requiring visits from the home nurse to assist her with medication management.

Joan doesn't trust the banks, and hides money around the house. She places a $50 note under her sofa cushion for safe‐keeping. Some days later, she looks ‘everywhere’ for this note, and can't find it.

From Joan's perspective, the only logical conclusion she can draw is that ‘somebody must have stolen it.’ Given that the home nurse is the only other person who has access to her home, it's a short step for her to conclude that the nurse is stealing her money.

This belief meets the technical definition of a delusion, but do any of us imagine that the prescription of an antipsychotic will modify this belief in any way?

Hallucinations are common in all forms of dementia. Although estimates vary considerably, their prevalence in Alzheimer's disease (AD) has been estimated at between 4% and 76%.^14^ A hallucination can be defined as a perception that occurs in the absence of a stimulus. We would strongly contend, on first principles, that it is very difficult to mount a case that such perceptual abnormalities can be merely ‘reactive’ or ‘responsive’ to anything other than organic changes within the brain. The fact that specific types of hallucinations occur much more frequently in certain dementia subtypes for example, visual hallucinations in dementia with Lewy bodies (DLB) and Parkinson's disease dementia (PDD) only speaks to further underscore their organic origins.

Psychotic symptoms may respond better to the therapeutic effect of antipsychotics in DLB compared to other dementias, perhaps due to reasons related to its unique underlying neuropathological trajectory and neurohormonal disturbances.^15^, ^16^ These mechanisms in DLB may also pose a greater risk with antipsychotic treatment compared to other dementias.^17^, ^18^

Where hallucinations are causing distress and significant decline in health, we argue it is frankly inhumane and unethical to deny appropriate treatment with antipsychotics. This is particularly relevant when there is a risk of harm to self or others (e.g., family or care staff), due to the presence of acute or severe psychotic symptoms. That is, the benefits of antipsychotics in alleviating these specific symptoms outweigh the risk of doing nothing about them. For example, uncontrolled episodes of psychosis may deny the individual basic human rights such as eating, sleeping and living well or at least in a decent manner. Notwithstanding this, it has been estimated that antipsychotic treatment is only appropriate in 10% of cases and such treatment causes seriously deleterious effects, such as falls, strokes and death.^19^

## 4 ORGANICITY AND TREATMENT OF NON‐PSYCHOTIC BPSD

Moving beyond the psychotic symptoms that arise as a result of dementia, certain other types of behaviours are more commonly found in specific dementia subtypes.^7^ Surely, if we are to accept that all behaviours are ‘reactive or responsive,’ this should not be the case?

The behavioural variant of frontotemporal dementia (bvFTD), for example, is characterised by an excess of behaviours that are understandable in the context of the known functions of the frontal lobes. Key amongst these are their role in maintaining emotional control, impulse control, attention and concentration, and insight. Issues that commonly arise with bvFTD often relate to an excess of explosive/impulsive, affectively labile and perseverative (repetitive motor or vocal) behaviours that are underpinned by disrupted brain function.^20^ This is not to deny that their expression is not influenced by environment, but the reality of their organicity cannot be ignored.

Depression is very common in all types of dementia, and the neurohormonal and neuroanatomical alterations in dementia lend further support, at least in part, to an organic origin. For example, the presence of white matter lesions and grey matter atrophy in brainstem tegmental nuclei in older adults with and without dementia is implicated in depression,^21^, ^22^ as is dorsolateral prefrontal cortical dysfunction.^23^ There is also a link between 5‐HT, or serotonin deficiency, and the development of cognitive deterioration with concomitant BPSD.^24^ Serotonergic system dysfunction can play a major role in emotional disturbances, including anxiety and depression. Further, there is some evidence that suggest 5‐HTR1A dysfunction occurs when depressive symptoms emerge in AD.^24^

Further, clarifying causes of depression in dementia is difficult and is complicated by the complexity of its diagnosis and treatment. Currently there is no validated diagnostic tool to diagnose depression, nor any international consensus regarding diagnostic criteria for major depression, for people with severe dementia. This difficulty is further compounded by the suggestion that antidepressants may be of a limited value for PLWD who are experiencing depression.^25^ A recent Cochrane review (2018) found that 12‐weeks of antidepressant therapy had little or no impact on depression rating scores when compared to placebo.^26^

Nonetheless, we contend that the above evidence, along with the possibility that depression may represent a behavioural prodrome of dementia, and that its prevalence may differ both qualitatively and quantitatively by dementia subtype (for example, vascular dementia [VaD] versus AD)^27^ makes the argument of depression being caused purely by unmet needs difficult to sustain.

## 5 HEALTH RISKS OF PSYCHOTROPIC TREATMENT

The medico‐ethical dilemmas in treatment decisions for BPSD should always be carefully evaluated before such decisions are made. Severe and life threatening BPSD, including hallucinations and delusions, should be first managed with person‐centred psychosocial approaches (e.g., distraction therapy) before any psychopharmacotherapy is initiated. If recommended, psychotropic prescribing for PLWD should be strictly time‐limited and carefully monitored, due to the high risk of serious adverse effects, such as stroke and death.^28^ The impact of such risk is much greater with a prolonged exposure and older age.^13^

Psychotropic‐induced excessive sedation (also known as ‘chemical restrain’) may result in falls and delirium.^29^, ^30^ Further, these medications increase the risk of hospitalisation and mortality.^31^, ^32^ For instance, Banerjee (2009) estimated that antipsychotics were responsible for about 1800 deaths and almost as many strokes each year in the UK.^33^ Similar trends were also found in other countries.^34^, ^35^ Psychotropic polypharmacy is also common in PLWD,^34^ which increases the risk of mortality by 41% for antipsychotic‐antidepressant‐benzodiazepine combination, and by 119% for antipsychotic‐benzodiazepine combination therapy.^36^

The risk of mortality and other serious adverse effects appear to be influenced by multiple factors including age, type and severity of dementia, neuropsychiatric burden, choice of psychotropic drug (e.g., typical vs. atypical antipsychotics) and dose, polypharmacy and comorbidities.^37^ For example, delusional beliefs and hallucinations may be hypersensitive to antipsychotic treatment and its adverse outcomes. Patients with DLB and PDD receiving antipsychotics (particularly atypical antipsychotics) may be more prone to a severe neuroleptic sensitivity reaction (NSR), which is characterised by the presence of extra‐pyramidal side effects such as rigidity and bradykinesia, and increased risk of mortality.^17^ It is postulated that NSR is caused by dopamine D2 receptor dysfunction.^18^

Thus, psychotropic medications including antipsychotics should only be used as the last resort when managing BPSD.

## 6 CONCLUSIONS

The rhetoric against using psychotropics for BPSD management on the basis that all behaviours are reactive or responsive lacks logic and the support of evidence. Although person‐centred psychosocial interventions should appropriately be used as the mainstay of treatment for BPSD, psychopharmacotherapy still has an important adjunctive role in managing certain BPSD, particularly in those subtypes/symptoms (e.g., DLB/hallucinations) with more organic, and/or less environmentally‐based, aetiology. In these instances, time‐limited prescribing with monitoring of therapeutic responses and adverse effects of these agents should be a priority, as they have limited efficacies and serious adverse outcomes. Although, it is not an exhaustive list, we provided a number of examples above that demonstrate the presence of organic aetiology in various BPSD within different dementias. Thus, it is important to consider the organicity of BPSD in therapeutic management plans. Despite what is posited herein, further research that involves developing and synthesizing high quality evidence, entailing rigorous study design (e.g., longitudinal studies on the impact of environmental factors on BPSD), valid setting‐specific and dementia (subtype)‐specific tools and large (epidemiological) samples with a varying severity of dementia is strongly needed. This would propound answers to help clinicians understand the complex overlap or interplay of reactive, responsive and/or organic causes of BPSD. In the absence of high‐quality evidence, a combination of evidence‐ and experience‐based medicine can be a useful approach to redress the existing gaps on how to address best the (in)appropriate use of psychotropic medications in PLWD, a population that often have multimorbidity and polypharmacy. The language used in describing certain BPSD may have profound implications on the way we treat them. Finally, it is worth noting that mislabelling or misattributing symptoms and/or subsequent treatment with inappropriate or prolonged therapies (e.g., psychotropic over‐prescribing) could be a matter of life and death in this frail population.

## CONFLICT OF INTEREST

The authors declared no potential conflicts of interest with respect to the research, authorship, and/or publication of this article.

## References

[1] Guideline Adaptation Committee . Clinical Practice Guidelines and Principles of Care for People with Dementia. Sydney: NHMRC Cognitive Decline Partnership Centre‐ Guideline Adaptation Committee; 2016.

[2] Atee M, Morris T, Macfarlane S, Cunningham C. Pain in dementia: prevalence and association with neuropsychiatric behaviors. J Pain Symptom Manag. 2020. In press. 10.1016/j.jpainsymman.2020.10.011 33068708

[3] Kozman MN, Wattis J, Curran S. Pharmacological management of behavioural and psychological disturbance in dementia. Hum Psychopharmacol Clin Exp. 2006;21:1‐12. 10.1002/hup.745 16389667

[4] Tible OP, Riese F, Savaskan E, von Gunten A. Best practice in the management of behavioural and psychological symptoms of dementia. Ther Adv Neurol Disord. 2017;10:297‐309. 28781611 10.1177/1756285617712979 PMC5518961

[5] Eriksson S. Impact of the environment on behavioral and psychological symptoms of dementia. Int Psychogeriatr. 2000;12:89.

[6] Lawlor BA. Environmental and social aspects of behavioral disturbances in dementia. Int Psychogeriatr. 1997;8:259‐261. 10.1017/s104161029700344x 9154573

[7] The International Psychogeriatric Association (IPA) . The IPA Complete Guides to Behavioral and Psychological Symptoms of Dementia (BPSD)‐ Specialists Guide. Northfield, Illinois; 2012.

[8] van der Linde RM, Dening T, Stephan BCM, Prina AM, Evans E, Brayne C. Longitudinal course of behavioural and psychological symptoms of dementia: systematic review. Br J Psychiatry. 2016;209:366‐377. 27491532 10.1192/bjp.bp.114.148403 PMC5100633

[9] Macfarlane S, Atee M, Morris T, et al. Evaluating the clinical impact of national dementia behaviour support programs on neuropsychiatric outcomes in Australia. Front Psychiatr. 2021;12:652254. https://www.frontiersin.org/articles/10.3389/fpsyt.2021.652254 10.3389/fpsyt.2021.652254 PMC8076549 33927656

[10] Harrison F, Cations M, Jessop T, et al. Prolonged use of antipsychotic medications in long‐term aged care in Australia: a snapshot from the HALT project. Int Psychogeriatr. 2020;32:335‐345. 31969207 10.1017/S1041610219002011

[11] Jost BC, Grossberg GT. The evolution of psychiatric symptoms in Alzheimer's disease: a natural history study. J Am Geriatrics Soc. 1996;44:1078‐1081. 10.1111/j.1532-5415.1996.tb02942.x 8790235

[12] Reus VI, Fochtmann LJ, Eyler AE, et al. The American Psychiatric Association practice guideline on the use of antipsychotics to treat agitation or psychosis in patients with dementia. Am J Psychiatr. 2016;173:543‐546. 27133416 10.1176/appi.ajp.2015.173501

[13] Bjerre LM, Farrell B, Hogel M, et al. Deprescribing antipsychotics for behavioural and psychological symptoms of dementia and insomnia: evidence‐based clinical practice guideline. Can Fam Physician. 2018;64:17‐27. 29358245 PMC5962971

[14] Bassiony MM, Lyketsos CG. Delusions and hallucinations in Alzheimer's disease: review of the brain decade. Psychosomatics. 2003;44:388‐401. 12954913 10.1176/appi.psy.44.5.388

[15] Samuel W, Caligiuri M, Galasko D, et al. Better cognitive and psychopathologic response to donepezil in patients prospectively diagnosed as dementia with Lewy bodies: a preliminary study. Int J Geriatr Psychiatr. 2000;15:794‐802. 10.1002/1099-1166(200009)15:9<794::aid-gps178>3.0.co;2-1 10984725

[16] Aarsland D, Perry R, Larsen JP, et al. Neuroleptic sensitivity in Parkinson's disease and parkinsonian dementias. J Clin psychiatry. 2005;66:633‐637. 15889951

[17] McKeith I, Fairbairn A, Perry R, Thompson P, Perry E. Neuroleptic sensitivity in patients with senile dementia of Lewy body type. BMJ. 1992;305:673‐678. 1356550 10.1136/bmj.305.6855.673 PMC1882909

[18] Piggott MA, Perry EK, Marshall EF, et al. Nigrostriatal dopaminergic activities in dementia with lewy bodies in relation to neuroleptic sensitivity: comparisons with Parkinson's disease. Biol Psychiatry. 1998;44:765‐774. 9798081 10.1016/s0006-3223(98)00127-9

[19] van der Spek K, Gerritsen DL, Smalbrugge M, et al. Only 10% of the psychotropic drug use for neuropsychiatric symptoms in patients with dementia is fully appropriate. The PROPER I‐study. Int Psychogeriatr. 2016;28:1589. 27587349 10.1017/S104161021600082X

[20] Piguet O, Hornberger M, Mioshi E, Hodges JR. Behavioural‐variant frontotemporal dementia: diagnosis, clinical staging, and management. Lancet Neurology. 2011;10:162‐172. 21147039 10.1016/S1474-4422(10)70299-4

[21] Makovac E, Serra L, Spanò B, et al. Different patterns of correlation between grey and white matter integrity account for behavioral and psychological symptoms in Alzheimer's disease. J Alzheimer's Dis. 2016;50:591‐604. 26836635 10.3233/JAD-150612

[22] Wilson RS, Nag S, Boyle PA, et al. Brainstem aminergic nuclei and late‐life depressive symptoms. JAMA Psychiatry. 2013;70:1320‐1328. 24132763 10.1001/jamapsychiatry.2013.2224 PMC3856195

[23] Terada S, Oshima E, Sato S, et al. Depressive symptoms and regional cerebral blood flow in Alzheimer's disease. Psychiatr Res Neuroimaging. 2014;221:86‐91. 10.1016/j.pscychresns.2013.11.002 24296273

[24] Chakraborty S, Lennon JC, Malkaram SA, Zeng Y, Fisher DW, Dong H. Serotonergic system, cognition, and BPSD in Alzheimer's disease. Neurosci Lett. 2019;704:36‐44. 30946928 10.1016/j.neulet.2019.03.050 PMC6594906

[25] Banerjee S, Hellier J, Dewey M, et al. Sertraline or mirtazapine for depression in dementia (HTA‐SADD): a randomised, multicentre, double‐blind, placebo‐controlled trial. Lancet. 2011;378:403‐411. 21764118 10.1016/S0140-6736(11)60830-1

[26] Dudas R, Malouf R, McCleery J, Dening T. Antidepressants for treating depression in dementia. Cochrane Database Syst Rev. 2018;8:CD003944. 10.1002/14651858.CD003944.pub2 30168578 PMC6513376

[27] Park JH, Lee SB, Lee TJ, et al. Depression in vascular dementia is quantitatively and qualitatively different from depression in Alzheimer's disease. Dement Geriatr Cognit Disord. 2007;23:67‐73. 17114882 10.1159/000097039

[28] Pink J, O’Brien J, Robinson L, Longson D. Dementia: assessment, management and support: summary of updated NICE guidance. BMJ. 2018;361:k2438. 10.1136/bmj.k2438 29925626

[29] Sterke C, Verhagen A, van Beeck E, van der Cammen T. The influence of drug use on fall incidents among nursing home residents: a systematic review. Int Psychogeriatr. 2008;20:890‐910. 18416875 10.1017/S104161020800714X

[30] Izza MAD, Lunt E, Gordon AL, Gladman JRF, Armstrong S, Logan P. Polypharmacy, benzodiazepines, and antidepressants, but not antipsychotics, are associated with increased falls risk in UK care home residents: a prospective multi‐centre study. Eur Geriatr Med. 2020;11:1043‐1050. 32813154 10.1007/s41999-020-00376-1 PMC7716922

[31] Zerah L, Boddaert J, Leperre‐Desplanques A, et al. Association between psychotropic and cardiovascular iatrogenic alerts and risk of hospitalizations in elderly people treated for dementia: a self‐controlled case series study based on the matching of 2 French health insurance databases. J Am Med Dir Assoc. 2017;18:549. ‐549. e513. 10.1016/j.jamda.2017.02.001 28330633

[32] Johnell K, Jonasdottir Bergman G, Fastbom J, Danielsson B, Borg N, Salmi P. Psychotropic drugs and the risk of fall injuries, hospitalisations and mortality among older adults. Int J Geriatr Psychiatr. 2017;32:414‐420. 10.1002/gps.4483 PMC5347947 27113813

[33] Banerjee S. The use of antipsychotic medication for people with dementia: time for action. A Report for the Minister of State for Care Services by Professor Sube Banerjee. London: The Stationary Office; 2009.

[34] Jester DJ, Molinari V, Zgibor JC, Volicer L. Prevalence of psychotropic polypharmacy in nursing home residents with dementia: a meta‐analysis. Int Psychogeriatr. 2021;1‐16. 10.1017/s1041610220004032 33407955

[35] Snowdon J, Galanos D, Vaswani D. Patterns of psychotropic medication use in nursing homes: surveys in Sydney, allowing comparisons over time and between countries. Int Psychogeriatr. 2011;23:1520. 21429281 10.1017/S1041610211000445

[36] Nørgaard A, Jensen‐Dahm C, Gasse C, Hansen ES, Waldemar G. Psychotropic polypharmacy in patients with dementia: prevalence and predictors. J Alzheimer's Dis. 2017;56:707‐716. 28035931 10.3233/JAD-160828

[37] Schwertner E, Secnik J, Garcia‐Ptacek S, et al. Antipsychotic treatment associated with increased mortality risk in patients with dementia. A registry‐based observational cohort study. J Am Med Dir Assoc. 2019;20:323‐329.e322. 30824220 10.1016/j.jamda.2018.12.019

